# ARTD1 regulates osteoclastogenesis and bone homeostasis by dampening NF-κB-dependent transcription of *IL-1β*

**DOI:** 10.1038/srep21131

**Published:** 2016-02-17

**Authors:** Agnieszka Robaszkiewicz, Chao Qu, Ewelina Wisnik, Tomasz Ploszaj, Ali Mirsaidi, Friedrich A. Kunze, Peter J. Richards, Paolo Cinelli, Gabriel Mbalaviele, Michael O. Hottiger

**Affiliations:** 1 Department of Molecular Mechanisms of Disease, University of Zurich, 8057 Zurich, Switzerland; 2 Department of Environmental Pollution Biophysics, University of Lodz, Pomorska 141/143, 90-236 Lodz, Poland; 3 Division of Bone and Mineral Diseases, Washington University School of Medicine, 660 South Euclid Avenue, Campus Box 8301, St. Louis, MO 63110; 4 Department of Molecular Biology, Medical University of Lodz, Narutowicza 60, 90-136 Lodz, Poland; 5 Competence Centre for Applied Biotechnology and Molecular Medicine, University of Zurich, 8057 Zurich, Switzerland; 6 Life Science Zurich Graduate School, Molecular Life Science Program, University of Zurich, 8057 Zurich, Switzerland; 7 Division of Trauma Surgery, Center for Clinical Research, University Hospital Zurich, 8091 Zurich, Switzerland

## Abstract

While ADP-ribosyltransferase diphtheria toxin-like 1 (ARTD1, formerly PARP1) and its enzymatic activity have been shown to be important for reprogramming and differentiation of cells, such as during adipogenesis, their role and mechanism in regulating osteoclastogenesis and bone homeostasis are largely unknown. Here, in cell culture-based RANKL-induced osteoclastogenesis models, we show that silencing of ARTD1 or inhibition of its enzymatic activity enhances osteoclast differentiation and function. As a consequence of ARTD1 silencing or inhibition, the recruitment of p65/RelA to the *IL-1β* promoter, which is associated with transcriptionally active histone marks, *IL-1β* expression and inflammasome-dependent secretion of IL-1β are enhanced. This subsequently promotes sustained induction of the transcription factor *Nfatc1/*A and osteoclastogenesis in an autocrine manner via the IL-1 receptor. *In vivo*, *Artd1*-deficient mice display significantly decreased bone mass as a consequence of increased osteoclast differentiation. Accordingly, the expression of osteoclast markers is enhanced in mutant compared to wild-type mice. Together, these results indicate that ARTD1 controls osteoclast development and bone remodelling via its enzymatic activity by modulating the epigenetic marks surrounding the *IL-1β* promoter and expression of *IL-1β* and subsequently also *Nfatc1/*A.

ADP-ribosyltransferase diphtheria toxin-like 1 (ARTD1, formerly called PARP1) belongs to the family of ADP-ribosyltransferases (ARTs) and utilizes NAD^+^ for the synthesis of ADP-ribose polymers on acceptor proteins. The human ARTD family consists of 18 proteins with either mono- or poly-ADP-ribosyltransferase activity that all share the same ARTD signature motif in their homologous catalytic domain. Several inhibitors have been developed that inhibit ARTD1 activity, but also other ARTD family members, because of their similarity and because they mostly mimic β-NAD^+^ 1. Since the discovery of ARTD1, most studies have focused on its role in DNA damage detection and repair responses^2^. However, over the past decade, ARTD1 has gained increasing attention for its role in gene regulation^3^,^4^,^5^,^6^,^7^. Previous studies have shown that ARTD1 is the now long forgotten RNA polymerase II basal transcription factor TFIIC, which represses nick-dependent transcription in biochemical assays^8^. The positive or negative impact of ARTD1 on gene expression results primarily from its cooperation with transcription factors such as NF-κB, and chromatin remodelling and modification^9^.

Accumulating data are now linking ARTD1 activity with cell differentiation and cell fate commitment. The change from preadipocytes to adipocytes triggered by the nuclear receptor PPARγ is a prime example of the role of nuclear ADP-ribosylation reactions in cellular differentiation. ARTD1-dependent PAR formation increases during differentiation of 3T3L1 preadipocytes and is required for sustained expression of PPARγ target genes^10^. Similarly, adipogenic differentiation is reduced in adipose-derived stromal cells isolated from *Artd1* knockout mice^11^. This function of ARTD1 is specific for adipogenesis, as osteogenic differentiation of adipose-derived stromal cells is not affected by *Artd1* deletion^10^,^11^. A recent study has shown that the methylcytosine dioxygenase ten-eleven translocation 1 (TET1) interacts with PPARγ in an ADP-ribosylation-dependent manner during adipogenesis^12^, suggesting a model of active, PAR-dependent DNA demethylation of key adipocyte-specific genes by TET1^12^. Moreover, ADP-ribosylation positively regulates TET1 expression by maintaining a permissive chromatin state at the gene locus.

Bone is a highly dynamic tissue that undergoes continuous remodelling, being dependent on an intricate array of factors, including cytokines/chemokines, hormones, and mechanical stimuli^13^,^14^. Normal bone turnover is maintained through the coordinated actions of osteoblasts and osteoclasts on bone formation and resorption respectively^15^. While osteoblasts develop from mesenchymal stem cells, osteoclasts are of myeloid origin^16^. The differentiation of osteoclast precursors (monocytes/macrophages) into fully functional osteoclasts depends on the stimulation of the receptor activator of nuclear factor kappa-B (NF-κB) (RANK) by its ligand (RANKL) and comprises of several steps: fate commitment, early phase multinucleation and late phase fusion with other committed cells, which finally gives rise to the large and functionally active bone-resorbing cells^17^.

At the molecular level, RANKL stimulation triggers the induction of the NF-κB heterodimer p65 (RelA)/p50 (NF-κB1), which induces the expression of nuclear factor of activated T-cells (*NFATc1*), a transcription factor that regulates the terminal RANKL-induced differentiation of osteoclasts^18^. NFATc1 drives the formation of bone-resorbing cells and regulates expression of osteoclast-specific genes such as that of cathepsin K (*Ctsk*) and tartrate-resistant acid phosphatase (*Tracp*)^18^. In addition, the RANKL-induced NF-κB pathway controls the transcription of a wide range of pro-inflammatory cytokines, chemokines and growth factors^19^. Some of these cytokines such as TNFα, IL-1, IL-17, IL-4, and IFNs have been well documented to modulate the maturation of functionally active osteoclasts in an autocrine feedback loop^20^,^21^.

Two recent papers have shown that ARTD1 promotes osteoblast differentiation of mesenchymal stem cells *in vitro*, suggesting a possible involvement of ARTD1 in bone remodelling^22^,^23^. Furthermore, ARTD1 has also been reported to repress the gene expression of bone-resorbing factors such as *Tracp* and the a3 isoform of V-ATPase subunit (*Tcirg1*) during RANKL-induced osteoclastogenesis^24^,^25^, suggesting that ARTD1 might also regulate osteoclastogenesis and thereby bone remodelling, although the detailed molecular mechanism by which ARTD1 is thought to regulate osteoclastogenesis and bone formation is unknown.

These findings and our observation that ARTD1 is a co-factor of NF-κB-dependent gene expression prompted us to elucidate the molecular mechanisms responsible for the involvement of ARTD1 in osteoclastogenesis and bone homeostasis. Here, we report that silencing or inhibition of *Artd1* epigenetically enhances RANKL-induced and NF-κB-dependent expression of *IL-1β* that subsequently drives *NFATc1*-dependent osteoclastogenesis. The epigenetic regulation of *IL-1β* expression strongly depends on the enzymatic activity of ARTD1. Furthermore, *Artd1*-deficiency impairs bone mass, which is likely due to alterations in osteoclastogenesis.

## Results

### ARTD1 activity represses RANKL-induced osteoclast differentiation

**Figure 1:**
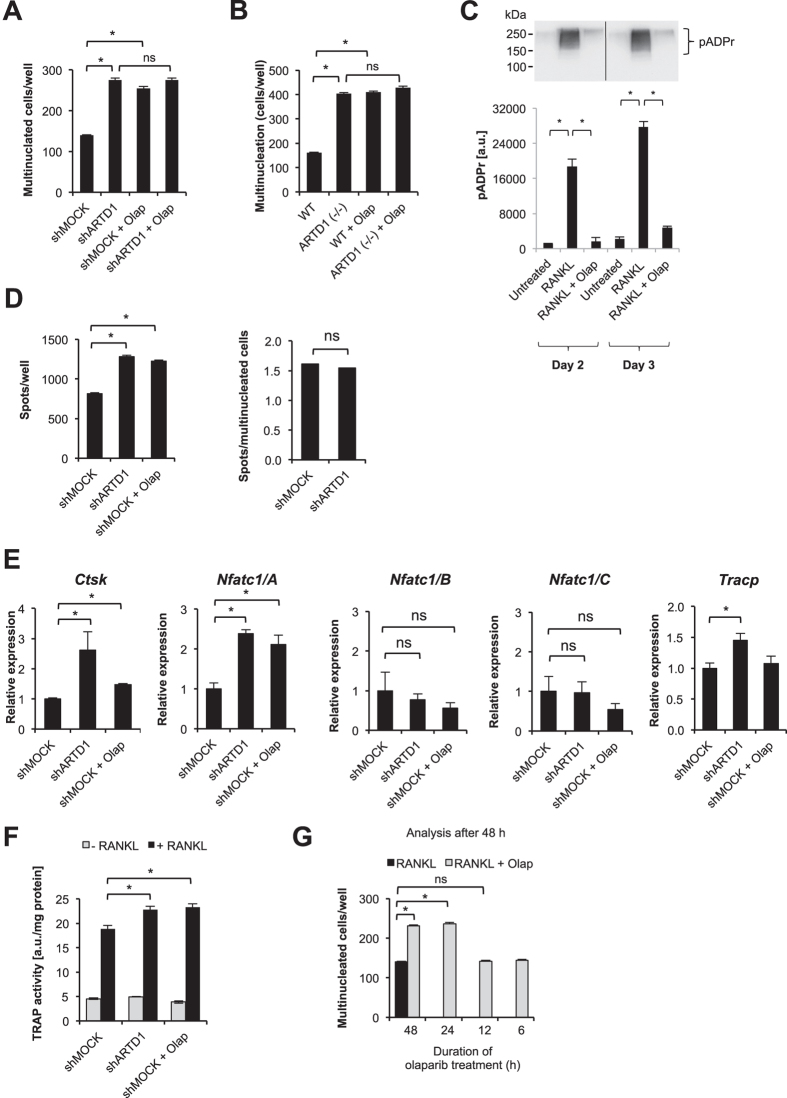
ARTD1 silencing or inhibition enhances osteoclast differentiation. The multinucleation capacity of (**A**) RAW264.7 macrophages expressing basal levels of *Artd1* (shMOCK) and *Artd1* knock-down (shARTD1), as well as of (**B**) bone marrow-derived macrophages (BMDM) isolated from wild-type (WT) and *Artd1*-deficient mice was quantified 48 h after RANKL (50 ng/ml) administration. The ADP-ribosylation inhibitor olaparib (1 μM) was added to shMOCK and WT BMDM cell cultures simultaneously with RANKL and was present throughout the course of differentiation. (**C**) The accumulation of ADP-ribosylated proteins in untreated BMDM (osteoclast precursors) and in 2 and 3 day old osteoclasts differentiated in the presence and absence of olaparib was studied with western blot. Densitometry was employed for quantification of ADP-ribosylation. (**D**) The resorbing activity of osteoclasts differentiated for 72 h in the presence of RANKL from RAW 264.7 cells with silenced and inhibited ARTD1 were determined based on matrix dissolution (left panel). The number of matrix dissolution spots was normalized to the number of multinucleated cells (right panel). (**E**) The gene expression of osteoclast markers was analysed in purified RAW 264.7-derived 3-day-old osteoclasts. (**F**) TRAP activity was quantified in RAW264.7 cells differentiated in the presence of RANKL for 48 h. (**G**) Olaparib was administered at selected time points after induction of osteoclastogenesis, and multinucleated cells were counted after 48 h.

To study the role of ARTD1 in osteoclastogenesis, RAW 264.7 macrophage-derived cells that overexpress an shRNA by retroviral transduction against a control sequence (shMock) or against *Artd1* (shARTD1) (Suppl. Fig. 1A,B) were stimulated with RANKL and allowed to differentiate into osteoclasts (see Suppl. Fig. 1C for overall experimental design). As expected, early multinucleation was observed in RAW 264.7 cells expressing shMock on day 2 (D2) after RANKL administration, and osteoclast expansion on day 3 (D3) (Suppl. Fig. 1C). Remarkably, silencing of *Artd1* in RAW 264.7 cells strongly enhanced the RANKL-induced number of multinucleated cells when compared to RANKL-stimulated shMOCK-expressing RAW 264.7 cells (Fig. 1A), indicating that ARTD1 represses osteoclastogenesis. The same experiment with stable knockdown of *p65* expression in RAW 264.7 cells caused reduced matrix dissolution and multinucleation (Suppl. Fig. 1 A,B,D), confirming the NF-κB-dependency of the system. An even more prominent effect of ARTD1 was found between RANKL-stimulated bone marrow-derived macrophages (BMDM) isolated from WT mice compared to *Artd1*-deficient (−/−) mice (Fig. 1B), confirming the results obtained with the engineered RAW 264.7 cells. RANKL treatment of shMock RAW 264.7 macrophages was associated with an induction of PAR formation on day 2 and 3 of differentiation (early development), which was inhibited by co-treatment of the cells with the ADP-ribosylation inhibitor olaparib (Fig. 1C). The presence of olaparib for the entire duration of osteoclast differentiation using shMOCK RAW 264.7 cells or WT BMDM phenocopied the enhanced multinucleation observed in RANKL-stimulated shARTD1-treated or *Artd1 (*−/−) macrophages (*cf.* Fig. 1A,B), indicating that ADP-ribosylation is important for repressing osteoclastogenesis. Since inhibition with olaparib did not further increase multinucleation in ARTD1-depleted or -deficient cells (Fig. 1A,B), the effect of olaparib on ARTD1-proficient cells was likely primarily due to inhibition of ARTD1. Multinucleated osteoclasts derived from *Artd1*-silenced or olaparib-treated RAW 264.7 macrophages were fully functional, as demonstrated by the dissolution of a mineral-coated surface (Fig. 1D, Suppl. Fig. 1E). At the molecular level, *Artd1* silencing or inhibition led to increased expression of the osteoclast marker gene *Nfatc1/A* (but not *Nfatc1/B* or *C*) (Fig. 1E), as well as increased TRAP enzymatic activity (Fig. 1F) 72 h after RANKL stimulation of RAW 264.7 macrophages, while transcription of *Ctsk* and *Tracp* was only affected by ARTD1 silencing and not inhibition.

To test at which time point during RANKL-induced multinucleation ARTD1 and its enzymatic activity are important, multinucleation was induced with RANKL for 48 (Fig. 1G) or 72 h (Suppl. Fig. 2A) and olaparib added at different time points following RANKL-treatment. A significant increase in multinucleation could be observed when olaparib was added in both settings from the beginning (48 h and 72 h, respectively) or for at least 24 h after RANKL treatment (24 h and 48 h respectively), while supplementation for a shorter time did not enhance multinucleation, suggesting that ARTD1 and its enzymatic activity enhance osteoclastogenesis during the time period between 24 and 48 h of differentiation, and not during the initial RANKL signalling (i.e. the first 24 h after RANKL treatment).

Together, these results demonstrate that ARTD1 and its enzymatic activity regulate RANKL-induced multinucleation and expression of the osteoclast differentiation driver NFATc1/A after initiation of the differentiation process and that the enhanced differentiated osteoclasts are fully functional.

### ARTD1 activity is induced during osteoclast differentiation in a topoisomerase II-dependent manner

Since our data indicated that the enzymatic activity of ARTD1 regulates osteoclastogenesis, we next investigated the mechanism by which the enzymatic activity of ARTD1 is induced. Since ROS have been described to strongly activate ARTD1^26^, we investigated whether the presence of the potent ROS scavenger N-acetylcysteine affect the differentiation potential of differentiating osteoclasts. However, no significant effects of N-acetylcysteine on cell multinucleation were observed at the concentration tested (Suppl. Fig. 3A). We have recently provided evidence that activation of ARTD1 and PAR formation during adipogenesis is dependent on topoisomerase II (topo II) activity^27^. Interestingly, treatment of RAW 264.7 cells during osteoclastogenesis with the topo II inhibitor merbarone enhanced multinucleation to the same level as observed with *Artd1* silencing (Suppl. Fig. 3B), suggesting that the observed ARTD1-mediated repression of cell multinucleation is regulated by topo II. The same observation was made with osteoclast progenitors from WT and *Artd1 (*−/−) mice. Treatment of progenitors with merbarone enhanced osteoclastogenesis in WT but not *Artd1 (*−/−) cells, suggesting that indeed topoisomerase activity induces ARTD1 activity, which subsequently represses osteoclastogenesis (Suppl. Fig. 3C).

### ARTD1 silencing or inhibition enhances osteoclastogenesis by stimulating IL-1β secretion and autocrine cell stimulation

**Figure 2:**
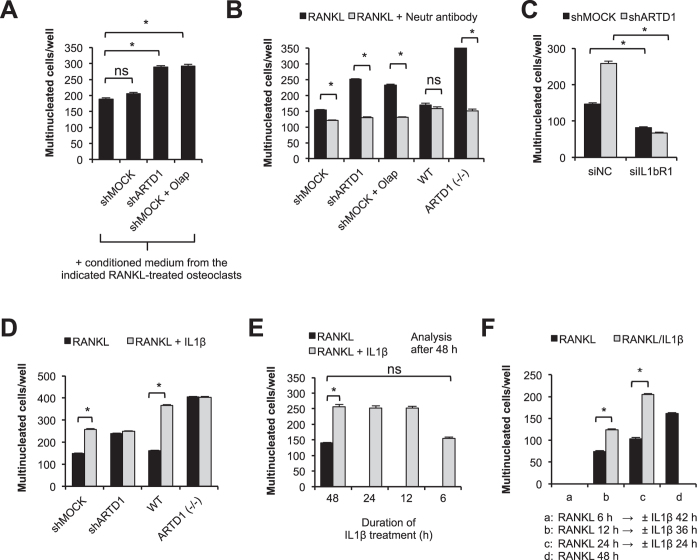
Inhibition of ADP-ribosylation as well as *Artd1* knock-out and knock-down are responsible for *IL-1β* overexpression-induced increase in osteoclast differentiation. (**A**) The pro-osteoclastogenic potential of media conditioned for 24 h by 3-day-old osteoclasts derived from RAW264.7 macrophages with normal ARTD1 levels as well as ARTD1 silenced and inhibited cells was compared. RANKL was added to the cell culture in combination with 30% conditioned medium and multinucleation was quantified after 48 h. (**B**) IL-1β neutralizing antibody (100 ng) was added every 8 h to RAW 264.7 and BMDM induced with RANKL for differentiation. (**C**) 24 h after transfection of shMOCK and shARTD1 RAW 264.7 macrophages with siRNA targeting IL-1β receptor 1 (*IL-1r1*) expression, cells were supplemented with RANKL and the role of IL-1R1 in the differentiation process was determined based on multinucleation. (**D**) To test the impact of IL-1β on osteoclastogenesis, RANKL was added to RAW 264.7 and BMDM cultured in the presence or absence of IL-1β (10 ng/ml) and the number of osteoclasts was counted on day 2. (**E**) IL-1β (2 ng/ml) was added at the indicated time points to shMOCK RAW 264.7 macrophages cultured in the presence of RANKL, and multinucleated cells were quantified 48 h after RANKL administration. (**F**) shMOCK RAW 264.7 macrophages were stimulated with RANKL for the indicated periods of time. RANKL was then washed away and the differentiation was allowed to continue for a total time of 48 h in the presence (grey bars) or absence (black bars) of IL-1β (10 ng/ml).

As autocrine or paracrine acting cytokines are known to regulate the differentiation of osteoclasts, we investigated whether ARTD1 controls osteoclastogensis via a secreted cytokine. Cultivation of RANKL-treated RAW264.7 macrophages with conditioned medium of either shARTD1 or olaparib-treated shMOCK, but not shMOCK osteoclasts, increased the multinucleation of RAW 264.7 macrophages through a secreted factor (Fig. 2A). Since IL-1β plays a major role in bone metabolism under physiological conditions^28^,^29^, we tested whether IL-1β could be involved in the enhanced multinucleation induced by ARTD1 depletion, inhibition or deficiency. Repeated addition of IL-1β neutralizing antibody to medium (every 8 h) from RANKL-induced *Artd1*-silenced or inhibited RAW 264.7 macrophages, as well as *Artd1 (*−/−) BMDM substantially reduced the ability of the medium to enhance multinucleation of RANKL-treated RAW264.7 macrophages (Fig. 2B), indicating that IL-1β is indeed the secreted factor responsible for the observed enhanced multinucleation of *Artd1 (*−/−), knock-downs or olaparib-treated cells. The transient silencing of *IL-1 receptor 1* (*IL-1r1*) in shMOCK or shARTD1 macrophages phenocopied the effect of IL-1β neutralizing antibody on the differentiation potential of macrophages (Fig. 2C, Suppl. Fig. 3D), further supporting the idea that the observed effect is mediated by IL-1β. Differentiation of RAW 264.7 and BMDM with a mixture of RANKL and recombinant IL-1β (10 ng/ml every 8 h^30^) led to an increase in the number of osteoclasts formed comparable to the level of RANKL-stimulated shARTD1-treated and *Artd1*-deficient macrophages, respectively (Fig. 2D). To further strengthen the importance of IL-1β in RANKL-induced osteoclastogenesis during the initial/sustained phase of osteoclastogenesis, shMOCK RAW264.7 macrophages were treated with either RANKL alone or RANKL together with IL-1β for different periods of times. RANKL alone was able to induce multinucleation only when added for at least 48 h, but not for a shorter time period (Fig. 2E). Addition of IL-1β at different time points to 48 h RANKL-treated macrophages significantly enhanced multinucleation for all tested time points, but 6 h of IL-1β (Fig. 2E), although analysis of the multinucleation for this sample after an additional 24 h (72 h after RANKL) also revealed significantly enhanced multinucleation (Suppl. Fig. 2B), suggesting that IL-1β is able to enhance RANKL-induced multinucleation throughout the analysed osteoclastogenesis, but required at least 12 h of incubation. Interestingly, the contribution of IL-1β to osteoclast formation was not limited to enhancement of multinucleation, since after a certain stage of osteoclast commitment (between 6 and 12 h after RANKL stimulation) and subsequent removal of RANKL, IL-1β drove osteoclast differentiation even in the absence of RANKL (Fig. 2F), indicating that IL-1β is important for sustaining osteoclastogenesis, once RANKL has initiated the differentiation process.

### Inflammasome activity is required for IL-1β secretion, and subsequent expression of the osteoclastogenic transcription factor NFATc1/A

**Figure 3:**
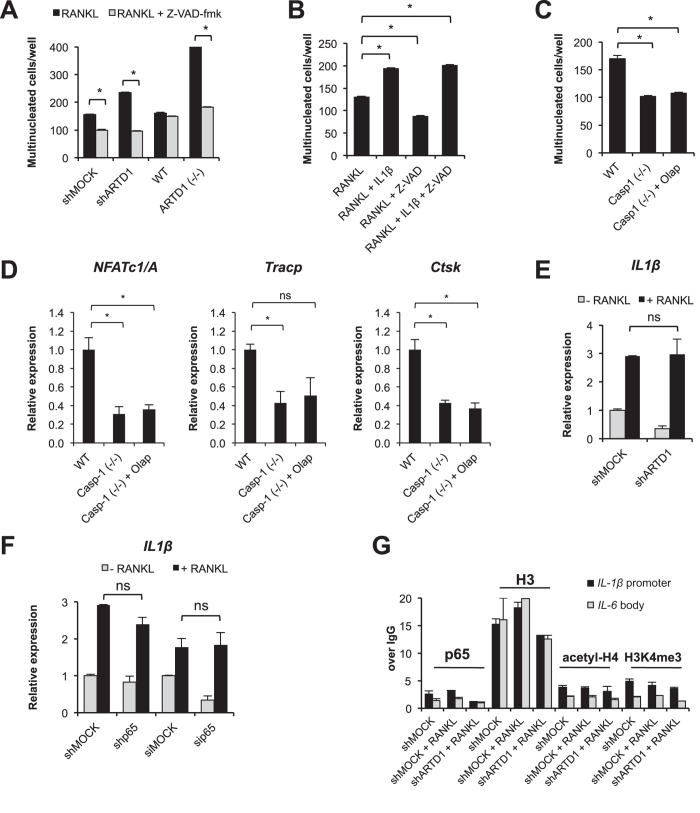
Inhibition of ADP-ribosylation as well as *Artd1* knock-out and knock-down are responsible for enhanced IL-1β expression during osteoclast differentiation. (**A**) The contribution of the inflammasome to osteoclast differentiation was studied by comparing cell multinucleation in RANKL-induced RAW 264.7 macrophage cultures supplemented or not with pan-caspase inhibitor Z-VAD-fmk (10 μM). (**B**) IL-1β (2 ng/ml) was added every 8 h to RANKL-induced RAW 264.7 macrophages (shMOCK) cultured in the presence or absence of Z-VAD-fmk. (**C**) The multinucleation of BMDM isolated from WT or *Casp-1*-deficient mice differentiated in the presence of RANKL and co-treated with olaparib or not for 2 d. (**D**) The expression of osteoclast markers in 3-day-old osteoclasts isolated from the RANKL-induced culture of WT, *Casp-1*-deficient BMDM and *Casp-1*-deficient BMDM incubated with olaparib was compared using real-time PCR. (**E**) *IL-1β* expression was compared between shMOCK and shARTD1 RAW264.7 macrophages stimulated with RANKL for 3 h. (**F**) The expression of *IL-1β* was compared between shMOCK or siMOCK RAW 264.7 macrophages and cells treated with shRNA or siRNA against *p65* after 3 h cell stimulation with RANKL. (**G**) The association of p65 with the promoter of *IL-1β* as well as H3 occupancy, H3K4 trimethylation and H4 acetylation was determined by ChIP in RAW 264.7 macrophages (shMOCK, shARTD1) incubated with RANKL for 1 h.

Caspase-1 is a subunit of the inflammasome complex, which cleaves pro-IL-1β, thereby causing the release of the active cytokine. We thus tested whether the pan-caspase inhibitor Z-VAD-fmk prevents the enhanced osteoclast formation observed in *Artd1*-silenced RAW 264.7 and *Artd1*-deficient BMDM (Fig. 3A). Indeed, Z-VAD-fmk added to the cell culture together with RANKL reduced the number of shARTD1-treated or *Artd1*-deficient osteoclasts to the level of shMOCK-treated and WT BMDM treated with caspase inhibitor, similar to the effect of IL-1β neutralizing antibody, indicating that caspase activity was required for osteoclast differentiation. IL-1β repeatedly added to the culture of differentiating cells was able to overcome the Z-VAD-fmk-induced repression of osteoclast formation (Fig. 3B), suggesting that caspase inhibition prevented processing of IL-1β. BMDM derived from *Casp-1*-deficient mice showed a reduced differentiation potential upon stimulation with RANKL when compared to WT BMDM, and very interestingly, olaparib failed to enhance osteoclast formation from *Casp-1*-deficient BMDM, further confirming that caspase-1 is indispensible for mediating the effect of ARTD1 silencing or inhibition on osteoclastogenesis (Fig. 3C). RANKL-stimulated *Casp-1*-deficient BMDM also expressed *Nfatc1/A, Tracp* and *Ctsk* mRNA to a significantly reduced level, which were not affected by treatment with olaparib (Fig. 3D), further supporting that the inflammasome-induced activation of caspase-1 and subsequent processing of IL-1β is required to enhance osteoclast differentiation due to ARTD1 deficiency or inhibition and stimulate the expression of *Nfatc1/A*.

### ARTD1 does not control expression of IL-1β upon initial RANKL signalling

To further elucidate the mechanism underlying the ARTD1 involvement in the regulation of *IL-1β* transcription during the differentiation of osteoclasts, we analysed the expression of *IL-1β* in RAW 264.7 macrophages after initial (3 h) RANKL stimulation (Fig. 3E). Although the level of *IL-1β* mRNA was increased 3 h after RANKL treatment, *Artd1* silencing had no effect on the expression of *IL-1β* in these cells. As the canonical NF-κB pathway is known to promote IL-1β production, we tested whether *IL-1β* expression is regulated by p65 during the initial RANKL-stimulation of RAW 264.7 macrophages. While RANKL stimulation for 3 h triggered *IL-1β* transcription, the level of *IL-1β* mRNA remained the same in shp65/sip65-silenced cells compared to the corresponding shMOCK/siMOCK-treated cells, suggesting that the initial RANKL-induced expression of *IL-1β* in macrophages is independent of the canonical NF-κB pathway (Fig. 3F). ChIP experiments confirmed a lack of p65 recruitment to the *IL-1β* promoter and a lack of chromatin remodelling within this sequence after short-term RANKL treatment (Fig. 3G). Furthermore, silencing of *Artd1* did not influence p65 binding, which agrees with our previous finding that ARTD1 does not regulate *IL-1β* transcription induced by short-term RANKL treatment (Fig. 3E).

### ARTD1 limits expression of IL-1β during sustained osteoclastogenesis by reducing NF-κB recruitment to the IL-1β promoter and maintaining a transcriptionally silent chromatin state around the p65-binding site

**Figure 4:**
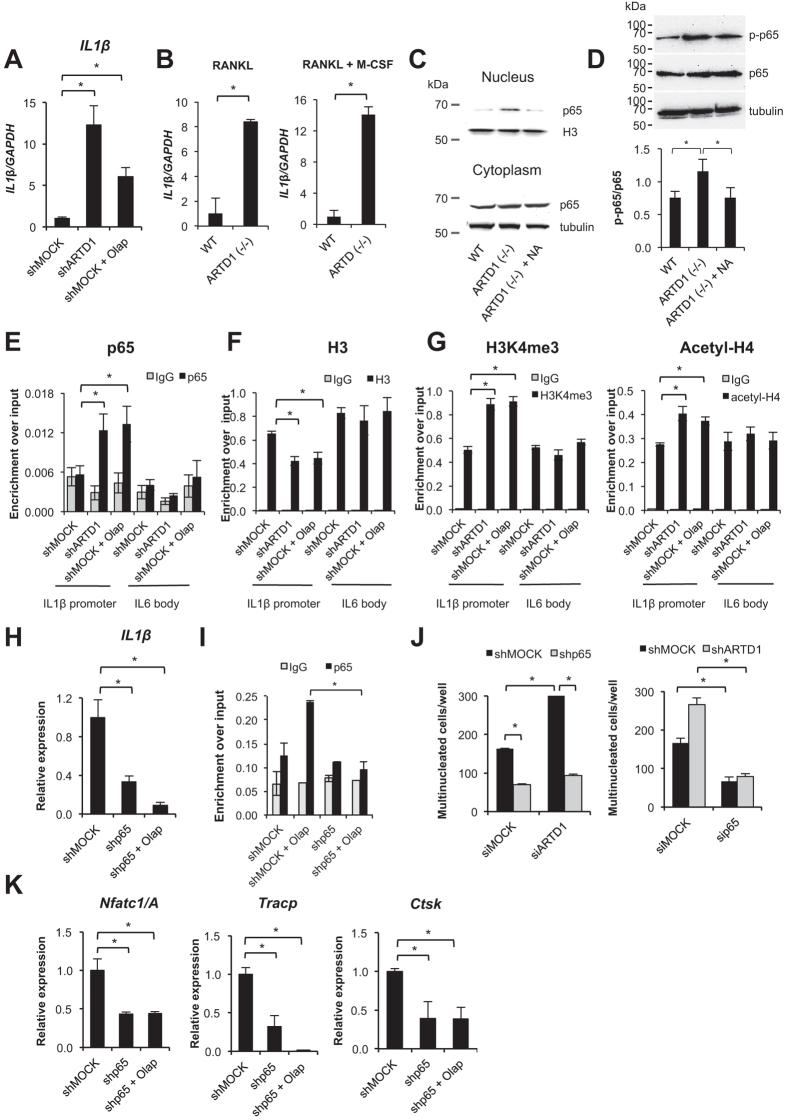
ARTD1 silencing or inhibition of ADP-ribosylation facilitates p65 recruitment to the promoter of *IL-1β* and chromatin remodelling at this site. (**A**) *IL-1β* expression was compared between 3-day-old osteoclasts derived from shMOCK and shARTD1 RAW264.7 macrophages. (**B**) The expression of IL-1β was quantified in 3-day-old osteoclasts derived from WT and *Artd1* (−/−) BMDM by real-time PCR. (**C,D**) The activation of NF-κB was analysed by measuring nuclear translocation (**C**) and Ser536 phosphorylation (**D**) of p65/RelA in 3-day-old osteoclasts derived from BMDM isolated from WT and *Artd1* (−/−) by western blotting. IL-1β neutralizing antibody (NA) was added to the medium of differentiating *Artd1* (−/−) every 8 h. (**E–G**) The association of p65 with the promoter of *IL-1β* as well as H3 occupancy, H3K4 trimethylation and H4 acetylation was determined by ChIP in RAW 264.7 macrophages-derived osteoclasts differentiated in the presence of RANKL for 72 h. As a negative control, the body of IL6 was used. (**H**) The expression of *IL-1β* was compared between 3-day-old osteoclasts derived from shMOCK and shp65 RAW 264.7 macrophages treated or not with olaparib. (**I**) Recruitment of p65 to the *IL-1β* promoter was assessed in 3-day-old osteoclasts derived from shMOCK and shp65 RAW 264.7 macrophages treated or not with olaparib. (**J**) The multinucleation of double-silenced RAW 264.7 macrophages (left panel: siARTD1/shp65, right panel: sip65/shARTD1) was quantified 48 h after induction of the differentiation with RANKL (72 h after cell transfection with siRNA). (**K**) The expression of osteoclast markers in 3-day-old shMOCK and shp65 ( ± olaparib) osteoclasts was quantified using real-time PCR.

In contrast to the above described short-term experiments (i.e., the initial RANKL-induced phase), ARTD1 silencing or inhibition significantly augmented *IL-1β* expression after 3 d of RANKL-induced osteoclastogenesis (Fig. 4A), suggesting that RANKL-induced osteoclast differentiation paves the way for ARTD1 to control the regulation of *IL-1β* expression. We also analysed the expression of *IL-1β* in differentiating osteoclast precursors isolated from *Artd1* (−/−) mice, confirming that *IL-1β* expression is indeed significantly upregulated in the absence of ARTD1 (Fig. 4B, left panel). This effect was independent of whether M-CSF was present during the RANKL-induced differentiation or not (Fig. 4B, right panel). To see whether the canonical NF-κB pathway is activated in 3 d differentiated osteoclasts that lack ARTD1, we analysed nuclear translocation of p65 by Western blot analysis. Indeed, p65 was increased in the nuclear extract of day 3 differentiated pre-osteoclasts derived from *Artd1* (−/−) mice compared to their WT counterparts (Fig. 4C). Furthermore, p65 phosphorylation at Ser536 was also enhanced in day 3 differentiated pre-osteoclasts derived from *Artd1* (−/−) mice compared to their WT counterparts (Fig. 4D). Addition of neutralizing IL-1β antibody reversed both the enhanced nuclear translocation of RelA and its phosphorylation at Ser536 in the cells from *Artd1* (−/−) mice (Fig. 4C,D). These results provide strong evidence that the observed enhanced RelA activation is driven by an autocrine (i.e. local) IL-1β loop.

Furthermore, ChIP experiments with ARTD1-silenced or inhibited cells revealed facilitated p65 recruitment to the *IL-1β* promoter in multinucleated cells (Fig. 4E), the eviction of histone H3 (Fig. 4F), as well as trimethylation of histone H3 and acetylation of H4 (Fig. 4G), histone marks of transcriptionally active chromatin. Stable silencing of *p65* resulted in decreased *IL-1β* expression in 3 d differentiated osteoclasts even in the presence of olaparib, indicating that during the sustained phase of differentiation, ARTD1-mediated ADP-ribosylation represses *IL-1*β transcription in osteoclasts in a p65-dependent manner (Fig. 4H). Furthermore, enhanced recruitment of p65 to the *IL-1β* promoter by olaparib treatment was inhibited in cells with stably knocked-down p65 (Fig. 4I). Interestingly, ARTD1 activity, but not the protein itself was required to control expression of *IL-1β*, because olaparib phenocopied the effect of ARTD1 without leading to dissociation of the enzyme from the chromatin (Suppl. Fig. 3F).

To confirm the contribution of canonical NF-κB signalling in the enhanced IL-1β-mediated osteoclast formation after 3 d by *Artd1*-silencing or inhibition, we compared the differentiation capacity between *Artd1* and *p65* single and double knock-downs (Fig. 4J, Suppl. Fig. 3G). The targeting of *Artd1* in shp65 RAW 264.7 macrophages with siRNA did not intensify the differentiation process, whereas silencing of *p65* dramatically reduced the osteoclastogenic potential of shARTD1 RAW 264 macrophages, suggesting that when p65 is knocked-down, ARTD1 does not repress osteoclastogenesis. Moreover, the presence of olaparib in differentiating shp65 osteoclasts had no influence on the differentiation process (Suppl. Fig. 2C). The change in osteoclast differentiation was also not observed in differentiating cells supplemented with conditioned medium from 3-d-old shp65 osteoclasts cultured in the presence or absence of olaparib (Suppl. Fig. 2D), providing further evidence that IL-1β is not released from *p65*-silenced osteoclasts even upon inhibition of ARTD1. Importantly, expression levels of the osteoclast markers *Nfatc1/A* (but not of *Nfatc1/B* and *Nfatc1/C*), *Ctsk* and *Tracp* were all dependent on p65, but remained unchanged after additional olaparib treatment of differentiating *p65*-silenced cells, further supporting that the presence of p65 is also required downstream of ARTD1-regulated *IL-1β* expression (Fig. 4K, Suppl. Fig. 3E).

### *Artd1*-deficient mice display a low bone mass phenotype owing to increased osteoclast differentiation

**Figure 5:**
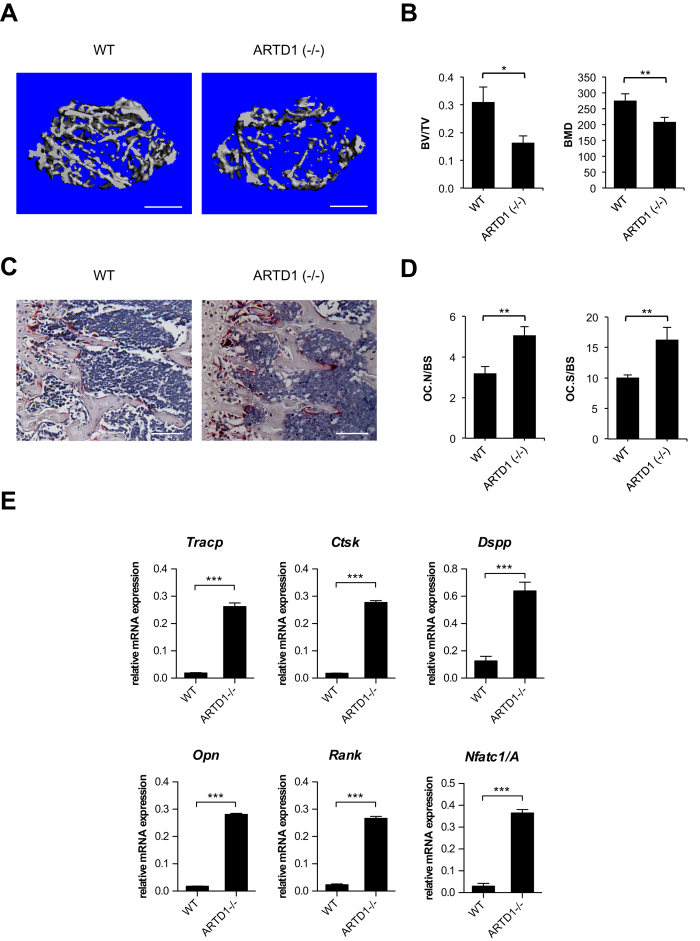
ARTD1 deficiency results in osteopenia and increased osteoclastogenesis. Femurs of 8-week-old *Artd1*-deficient (−/−) mice (n = 4) and their wild-type littermates (n = 4) were analysed by μCT. (**A**) Representative 3D reconstruction. Size bars = 500 μm. (**B**) Bone volume density (BV/TV) and bone mineral density (BMD) were quantified. (**C**) TRAP staining of tibial sections. Size bars = 100 μm. (**D**) Number of osteoclast (stained in red)/bone surface (Oc.N/BS) and osteoclast surface/bone surface (Oc.S/BS) were quantified. Values are mean ± SD. **p < 0.01. (**E**) Expression profiles of osteoclastogenic markers were analysed by qRT-PCR of RNA isolated from the proximal tibial epiphysis and part of the diaphysis of wt (n = 4) and *Artd1 (*−/−) (n = 5).

Our cell culture experiments provided strong evidence that ARTD1 represses the sustained phase of osteoclastogenesis. Mice lacking ARTD1 are thus to be expected to have altered bone structure due to enhanced osteoclast activity. To investigate whether ARTD1 indeed regulates bone homeostasis *in vivo*, long bones of age- and sex-matched wild-type (WT) and *Artd1*-deficient (−/−) mice were collected and analysed by micro-CT and histology. At 8 weeks of age, male *Artd1 (*−/−) mice exhibited a significant decrease in bone mass (BV/TV, Fig. 5A,B), along with decreases in bone mineral density (BMD, Fig. 5B), trabecular number (Tb.N) and a significant increase in trabecular spacing (Tb.Sp) (Suppl. Fig. 4A). Also the cortical envelope is osteopenic in *Artd1 (*−/−) mice compared to WT littermates (Suppl. Fig. 4B). The abnormal bone phenotype in *Artd1 (*−/−) mice is likely due to alterations in osteoclastogenesis, since the number of osteoclasts is significantly increased in mutant mice (Fig. 5C,D) while the number of osteoblasts was comparable between both mouse strains (Suppl. Fig. 4C). To strengthen the notion that the altered trabecular structure is the result of increased osteoclast differentiation, we analysed the expression levels of osteoclast specific gene markers in femurs (Fig. 5E). Indeed, the expression levels of *Tracp, Ctsk*, and *Rank* were all significantly upregulated in the bones of *Artd1 (*−/−) mice. The expression of the dentin sialophosphoprotein gene *Dspp*, the osteopontin gene *Opn*, and *Nfatc1/A* was also upregulated in *Artd1 (*−/−) mice (Fig. 1E), strongly supporting that the observed bone phenotype in *Artd1 (*−/−) mice is due to enhanced osteoclast formation.

## Discussion

**Figure 6:**
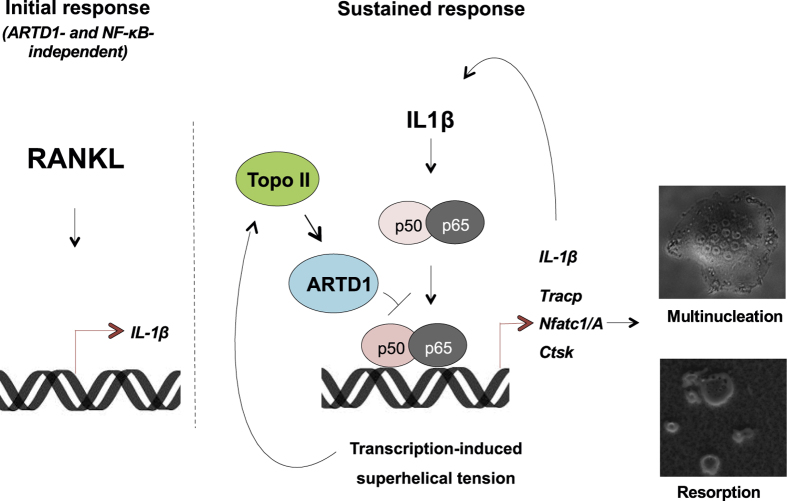
Graphical representation of the functional role of ARTD1 in RANKL-induced osteoclastogenesis.

Osteoclast deregulation is associated with several diseases, either through deficient osteoclast function, resulting in osteopetrosis^31^, or aberrant hyperactivation, giving rise to generalized bone loss as observed in osteoporosis^32^ and rheumatoid arthritis^33^. Moreover, osteoclasts cause bone complications in cancers, including multiple myeloma^34^, prostate and breast cancers^35^. Thus, understanding the molecular mechanisms that govern osteoclast differentiation and function is of major importance in the development of more effective therapeutic strategies to combat these diseases. The differentiation of osteoclasts from peripheral blood macrophages in cell culture allows studying osteoclastogenesis in great detail^36^. Our results show that ARTD1 regulates NF-κB-induced *IL-1β* expression in osteoclasts, leading to autocrine regulation of the expression of osteoclast markers (e.g. NFATc1/A). This is achieved through ARTD1’s ability to enzymatically alter the epigenetic status of the *IL-1β* promoter during sustained osteoclastogenesis. Thus, ARTD1 through the regulation of *IL-1β* expression has the ability to affect bone homeostasis (Fig. 6).

IL-1β is a multifunctional cytokine that regulates various cellular tissue functions including osteoclastogenesis^37^,^38^. In our analysis, *IL-1β* was initially induced in a RANKL-dependent but NF-κB-independent manner when tested after 3 h after RANKL treatment. It remains to be investigated, through which signalling cascade and transcription factor the (short term) expression of *IL-1β* is initiated. Besides canonical NF-κB, RANKL binding to its surface receptor also activates non-canonical NF-κB, AP1 and NFATc1^39^. All these factors possess the consensus sequence for the interaction with the *IL-1β* promoter, and may therefore be responsible for the observed expression of this cytokine. Moreover, the lack of promoter responsiveness to RANKL stimulation may be a result of the type of osteoclast precursors employed in this study. Bone marrow-derived macrophages require alternative cell polarization with M-CSF, which directs them toward anti-inflammatory responses, while RAW 234.7 macrophages express surface markers of type 2 macrophages (M2)^39^. Therefore, the differentiation process associated with extensive chromatin dynamics may prime *IL-1β* regulatory elements to effective *IL-1β* transcription, as well as place the ADP-ribosylation process in the centre of *IL-1β* expression regulatory events. In contrast, RANKL treatment for 3 days revealed that ARTD1 fully regulates *IL-1β* expression at this later time point in a NF-κB-independent manner, suggesting that RANKL induces IL-1β, which subsequently, in an autocrine loop (local manner), sustains its expression in an NF-κB- and ARTD1-dependent manner. IL-1β was able to enhance RANKL-induced multinucleation, but its action required at least 12 h of incubation. Interestingly, the contribution of IL-1β to osteoclast formation was not limited to enhancement of multinucleation, since after a certain stage of osteoclast commitment (between 6 and 12 h after RANKL stimulation) and subsequent removal of RANKL, IL-1β drove osteoclast differentiation even in the absence of RANKL.

Under physiological conditions, osteoclastogenesis is thought to be dependent on M-CSF and RANKL. However, IL-1β has been reported to induce osteoclast formation directly from M-CSF-induced osteoclast precursors overexpressing *c-Fos* or *Nfatc1*^40^, thus supporting the important function of IL-1β during osteoclastogenesis. Similarly, overexpression of *type 1 IL-1R* in BMDM is sufficient to drive osteoclastogenesis when RANKL is neutralized with a RANK–Fc fusion protein, suggesting that IL-1β can also act independently of RANKL^30^,^41^. Indeed, our studies indicate that IL-1β is important for sustaining osteoclastogenesis in an autocrine manner by inducing the sustained expression levels of important osteoclast markers such as NFATc1/A. The ARTD inhibitor olaparib did not completely phenocopy the effect of ARTD1 silencing on *Ctsk* and *Tracp,* as it did in the case of *Nfatc1/A*, but rather corresponded to *IL1β* expression, which is more strongly increased in shARTD1 than in shMOCK + Olap. Moreover, expression of these genes in differentiating osteoclasts is controlled by the RANKL–IL1β–p65 axis, as shown in Figs 3D and 4K. Therefore, their transcription is affected indirectly by ARTD1 and the p65–IL1β–p65 autocrine feedback loop. The olaparib-induced increase in *IL1β* expression is sufficient to achieve maximum osteoclast differentiation, although concomitant *Nfatc1/A* transcription might not be sufficient to augment *Tracp* and *Ctsk* expression.

The importance of IL-1β is further underlined by the observation that *IL-1β*-deficient mononuclear cells have a decreased RANKL responsiveness, although the response to TNF-α is maintained^42^,^43^. Molecular characterization of osteoclast precursors revealed a decreased expression of RANK as the underlying cause for a blunted response of *IL-1β*-deficient cells to RANKL, whereas other surface and functional markers such as c-fms, cathepsin K, TRAP, and MMP9 were similarly expressed.

Since olaparib also inhibits other ARTD family members, we cannot per se exclude that other ARTDs also contribute to osteoclastogensis. However, co-treatment of shARTD1-treated or *Artd1*-deficient cells with RANKL and olaparib did not cause a further increase in the number of osteoclasts developed, strongly suggesting that the inhibitory effect is primarily due to inhibition of the enzymatic activity of ARTD1. Our experiments using the topo II inhibitor merbarone suggest that topo II is responsible for the activation of ARTD1 during IL-1β-induced/sustained osteoclastogenesis, leading to the repression of fully functional osteoclast formation. The differentiation process is accompanied by extensive transcription of genes contributing to conditioning of osteoclast function and phenotype. The transcription machinery requires topo II activity, which may activate ARTD1, placing topo II upstream of ARTD1. A comparable mode of action has already been described for adipogenesis^27^, although the actual molecular mechanism remains to be elucidated. However, it is most likely that the enzymatic activity of topo II and the subsequent structural changes of the chromatin contribute to this process. In our *in vitro* model of osteoclast differentiation, topo II serves as a major component of the transcription–differentiation–transcription feedback loop, as transcription-induced DNA gyrase activity represses transcription of osteoclastogenic genes by activating ARTD1. Although investigating topo II recruitment to the *IL-1β* promoters in osteoclasts would be interesting, the lack of suitable antibodies currently prevents this type of analysis.

The impact of ARTD1 on gene expression remains mostly undefined, as its function is cell type-specific and depends e.g. on the local histone variants, interacting partner and ADP-ribosylation acceptor protein, and may therefore differ after differentiation. Moreover, ARTD1 has recently been shown to function in various aspects of the transcription process through a variety of mechanisms, including roles as a modulator of chromatin and a regulator of DNA methylation^4^,^5^. PARylation of KDM5B, a histone lysine demethylase that acts on H3 lysine 4 trimethyl (H3K4me3), has been shown to block the binding of KDM5B to chromatin and inhibit its demethylase activity^44^. Our results are thus in line with recent data showing that inhibition of the enzymatic activity of ARTD1 regulates the activity of KDM5^44^. It remains to be elucidated whether ARTD1 auto-modification is sufficient to promote these changes, or whether other proteins (histone modifiers or histone themselves) are also modified.

Of note, we cannot preclude that ARTD1 itself acts as a cofactor in the transcription machinery assembled with other transcription factors activated upon RANK stimulation, such as NFATc1 or AP1 independent of its enzymatic activity; however, this requires further analysis. Moreover, our results provide evidence that the inflammasome NOD-like receptor (NLR) family, pyrin domain-containing 3 (NLRP3) is activated upon RANKL stimulation to process pre-IL-1β, which is followed by subsequent secretion of IL-1β, although an inflammasome-independent source of IL-1β has already been described to also provoke inflammation and osteolytic bone disease^45^. Based on our results, inflammasome-dependent IL-1β would signal to the osteoclast in an autocrine manner, which is consistent with previous findings that osteoclasts express IL-1β receptor^46^. Restriction of an activating D301N mutation in Nlrp3 in myeloid cells of mice, induced systemic inflammation and severe osteopenia similar to mice globally expressing the knock-in mutation^47^,^48^. Mice expressing D301N only in osteoclasts show the same phenotype, due to increased osteolysis, without changing the number of osteoclasts or inducing systemic inflammation. Aside from its role in IL-1β maturation, Nlrp3^D301N^ expression enhances osteoclast bone resorbing ability through reorganization of the actin cytoskeleton, while promoting the degradation of ARTD1 at the same time. Thus, NLRP3 inflammasome activation is not restricted to the production of pro-inflammatory mediators only, but also leads to intracellular autonomous responses.

Interestingly, analysis of the bones from *Artd1* (−/−) mice showed a severely decreased BV/TV as a result of a decreased bone mineral density (BMD), number of trabeculae (Tb.N) and increased trabecular space (Tb.Sp). The cortical envelope in *Artd1* (−/−) mice was also osteopenic compared to wild-type littermates. Histological analysis of the bone samples by histology revealed the low bone mass phenotype is likely caused by deregulated osteoclastogenesis, since the number of osteoclasts is increased in mutant mice, while that of osteoblasts is unaffected, strongly supporting that the observed bone phenotype in *Artd1* (−/−) mice is due to enhanced osteoclast formation and activity.

Taken together, our results provide important novel insights into the mechanisms underlying the fine-tuning of the transcriptional processes involved in macrophage-to-osteoclast differentiation. Most importantly, ARTD1 and its enzymatic activity repress the expression of osteoclastogenesis-driving genes, including several osteoclast-specific genes through controlling the expression of *IL-1β*. Secondly, our results reinforce the essential function of ARTD1’s enzymatic activity for the role of NF-κB as a direct regulator of osteoclast formation. ARTD1 and its enzymatic activity thus keep the detrimental expression of *IL-1β* in osteoclasts in check by altering the chromatin structure and subsequently the accessibility of transcription factors such as NF-κB.

## Materials and Methods

### Antibodies and reagents

p65/RelA antibody (sc-372), α-tubulin antibody (H-300, sc-5546), and IgG (sc-2027) was from Santa Cruz Biotechnology, PARP1 antibody, histone H3 antibody (D1H2, #4499), and Cell Fractionation Kit (#9038) from Cell Signaling Technology, growth media, FBS, antibiotics, and Lipofectamine RNAiMAX from Life Technologies, IL-1β, IL6, TNFα and Z-VAD-fmk from Sigma Aldrich, H3K4me3 and acetyl-H4 antibody from Millipore, H3 antibody from Abcam, RANKL from Peprotech, siRNA from Qiagen. Corning Osteo Assay plates were from Corning. IL-1β neutralizing antibody was from R&D.

### Animals

Male *Artd1*-deficient C57BL/6J mice and aged-matched wild-type littermates were sacrificed at 6 and 8 weeks of age for retrieving bones for BMDM isolation and bone structure analysis, respectively. The *Artd1*-deficient mice were originally kindly provided by Zhao-Qi Wang^49^, and all mice were bred at the animal facility of the University of Zurich. We have focused on male C57BL/6J mice, because they exhibit higher bone mass (BV/TV) at baseline than female mice. All animal experiments were carried out in accordance with the Swiss and EU ethical guidelines and have been approved by the local animal experimentation committee of the Canton of Zurich under license #2012207 and following the 3R guidelines.

### Isolation of bone marrow-derived macrophages (BMDM)

Bone marrow was isolated from femurs and tibiae of 6-week-old male mice and after lysis of erythrocytes, cells were cultivated for one week in RPMI supplemented with 10% heat inactivated FBS, antibiotics (50 U/ml penicillin, 50 μg/ml streptomycin) and 20% M-CSF-containing medium conditioned by L929 fibroblasts. Over the following two weeks conditioned medium free RPMI was gradually replaced by DMEM.

### Cell culture condition

Both RAW 264.7 and BMDM were cultured in DMEM supplemented with 10% FBS, 50 U/ml penicillin, 50 μg/ml streptomycin, and 1% L-glutamine (growth medium) at 37°C in a humidified atmosphere of 5% CO_2_.

### Osteoclast differentiation

The differentiation of BMDM and RAW 264.7 macrophages was initiated by the addition of 50 ng/ml RANKL one day after cell seeding. For osteoclast quantification and determination of TRAP activity at the earliest possible time point, cells were plated at the density of 8,100 per cm^2^ and analysed 48 h after supplementation of the cell culture with RANKL (D2). For all other experiments, which required more osteoclasts, cells were seeded at the density of 32,400 per cm^2^ and analysed 72 h after RANKL administration (D3). The undifferentiated cells were removed from strongly attached osteoclasts by successive washing with DMEM (1x) and PBS (3x).

### Osteoclast formation and determination of their activity

Two-day-old cultures of differentiating cells were stained for TRAP activity using Acid Phosphatase, Leukocyte (TRAP) Kit (Sigma Aldrich) according to the manufacturer’s instructions. TRAP-positive, multinucleated cells (carrying 3 ≥ nuclei) were counted as osteoclasts and data shown represent the number of multinucleated cells derived from initially 2,500 bone marrow or RAW 264.7-derived macrophages stimulated with RANKL.

For quantification of TRAP activity, stained cells were dissolved in DMSO, solution was acidified with 3% HCl, absorbance was measured at 540 nm and normalized to the protein content, which was quantified with Lowry assay. Osteoclast resorbing activity was evaluated in the culture of cells differentiated for 3 days by counting matrix dissolution spots on the Corning® Osteo Assay plates (Corning Life Sciences). Undifferentiated cells were removed by washing and remaining osteoclasts were trypsinised for 15 min and washed off. After drying, images of plate surface were acquired on an Olympus inverted microscope.

### Generation of stable ARTD1 and p65 knock-downs and siRNA transfection

shRNA constructs specific for *ARTD1* and for *p65/RelA* (see Suppl. Table 1) in pSUPER retroviral vector were packed in HEK-293cell line into invasive viruses. RAW 264.7 macrophages were infected with generated retroviruses for 8 h. After another 48 h, puromycin (5 μg/ml) was added to select the transduced cells. For transient silencing *Artd1* and *p65* were targeted with siRNA using Lipofectamine RNAiMAX as a carrier. Efficiency of the silencing was monitored by Western blot analysis and real-time PCR. In order to analyse the impact of ARTD1 and p65 in osteoclast development, RANKL was added to cells 24 h after their transfection with siRNA and multinucleation was quantified on day 2 (D2).

### Gene expression

RNA was extracted with TRI Reagent, reversed transcribed (High Capacity cDNA Reverse Transcription Kit, Life Technologies) and cDNA was quantified by real-time PCR (Rotor-Gene 3000, Qiagen) using primers listed in Suppl. Table 1 and SYBR Green (Kapa Biosystems). *Gapdh* was used as a housekeeping control gene.

### Chromatin immunoprecipitation

Recruitment of p65 to promoter sequences, H3 and ARTD1 occupation as well as H3K4 trimethylation and H4 acetylation was studied with ChIP according to^50^. In brief, cells were cross-linked with 1% formaldehyde solution, isolated chromatin was sheared with the Bioruptor (Diagenode) and after incubation with antibody-conjugated protein G magnetic Dynabeads (Life Technologies), chromatin was isolated with phenol:chlorophorm:isoamyl alcohol. Extracted DNA was further analysed with real-time PCR. The list of primers used for IL-1β promoter and IL6 body are listed in Suppl. Table 1.

### Isolation of RNA from mouse bone

Total RNA was isolated from a region encompassing the proximal tibial epiphysis and part of the diaphysis of WT (n = 4) and *Artd1*^−/−^ (n = 5) mice using TRIzol reagent, and treated with TURBO DNase (Life Technologies) as previously described^51^.

### Bone mass and microstructure

Femurs from 8-week-old male mice were stabilized in 2% agarose gel, and analysed by micro-computed tomography (μCT) system (μCT 40; Scanco Medical AG, Zurich, Switzerland) as described previously^48^. The volume of interest analysed was located just proximal to the growth plate, spanning a height of 350 μm each for the metaphyseal region.

### Histology and histomorphometry

Tibiae were fixed in 10% formalin, decalcified in 14% (w/v) EDTA pH 7.2 for 10–14 days at room temperature, embedded in paraffin, sectioned at 5 μm thickness and mounted on glass slides. Sections stained with haematoxylin and eosin or for TRAP were used for the analysis of osteoblasts and osteoclasts, respectively, as described previously^48^. Photographs were taken and cell number counted using nanozoomer (Hamamatsu).

### Statistical analysis

Bars in the figures represent mean ± standard error of the mean (SEM). Student’s t-test was used to compare the difference between two means. *P < 0.05, **P < 0.01, ***P < 0.001. Results of the *in vitro* model of differentiation represent data from three independent biological replicates (with three technical replicates each).

## Additional Information

**How to cite this article**: Robaszkiewicz, A. *et al*. ARTD1 regulates osteoclastogenesis and bone homeostasis by dampening NF-κB-dependent transcription of *IL-1β*. *Sci. Rep.* **6**, 21131; doi: 10.1038/srep21131 (2016).

## Supplementary Material

Supplementary Information
