# Supplementary material for: ARTD1 regulates osteoclastogenesis and bone homeostasis by dampening NF-κB-dependent transcription of IL-1β
Source: Sci Rep. 2016 Feb 17;6:21131. doi: 10.1038/srep21131 (PMC4756713; doi:10.1038/srep21131)
Supplement: Supplementary Information [file srep21131-s1.pdf]

ARTD1 regulates osteoclastogenesis and bone homeostasis by dampening NF-κB-dependent transcription of *IL-1β*

Agnieszka Robaszkiewicz[1,2], Chao Qu[3], Ewelina Wisnik[2], Tomasz Ploszaj[4], Ali Mirsaidi[5], Friedrich A. Kunze[1,6], Peter J. Richards[5], Paolo Cinelli[7], Gabriel Mbalaviele[3] and Michael O. Hottiger[1,5,*]

Supplementary Figures and Table

The following materials are available in the online version of this article:

Figure S1. Efficiencies of stable knock-downs, experimental design of in vitro differentiation model, and functional osteoclast activity in knock-down cells.

Figure S2. Timing of olaparib or IL-1β effect on multinucleation, and contribution of p65 on olaparib effect on multinucleation.

Figure S3. Effect of NAC or merbarone on enhanced multinucleation by ARTD depletion/deletion.

Figure S4. ARTD1 deficiency causes osteopenia without changes in osteoblast parameters.

Table S1. List of real-time PCR and ChIP primer sequences.

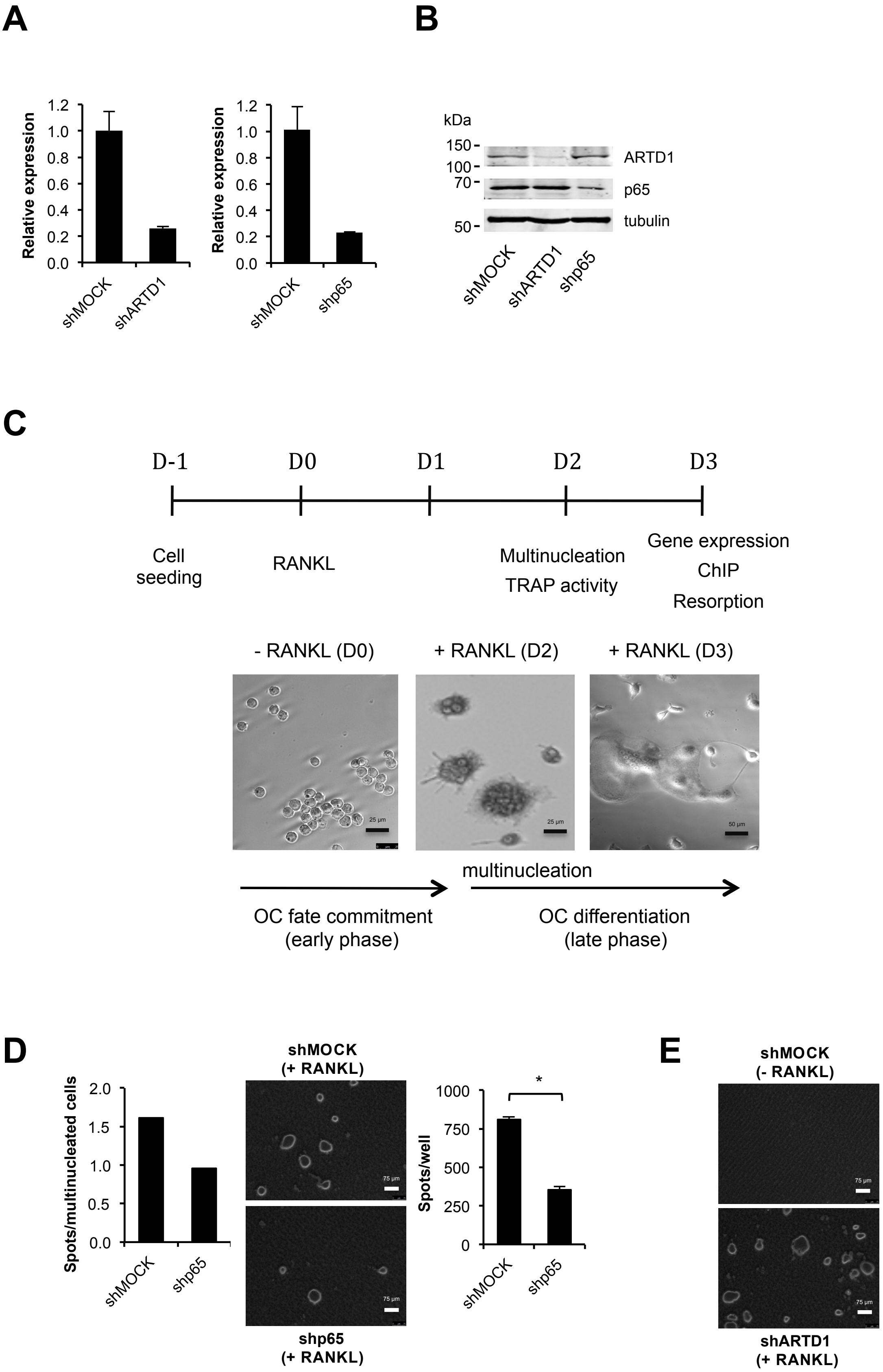

A
B
Relative expression
1.2
1.0
0.8
0.6
0.4
0.2
0.0
shMOCK
shARTD1
shp65
kDa
150
100
70
50
ARTD1
p65
tubulin
C
D-1
D0
D1
D2
D3
Cell seeding
RANKL
Multinucleation
TRAP activity
Gene expression
ChIP
Resorption
- RANKL (D0)
+ RANKL (D2)
+ RANKL (D3)
25 µm
50 µm
multinucleation
OC fate commitment (early phase)
OC differentiation (late phase)
D
Spots/multinucleated cells
2.0
1.5
1.0
0.5
0.0
shMOCK (+ RANKL)
shp65 (+ RANKL)
75 µm
Spots/well
1000
750
500
250
0
*
E
shMOCK (- RANKL)
shARTD1 (+ RANKL)

Figure S1. Efficiencies of stable knock-downs, experimental design of in vitro differentiation model, and functional osteoclast activity in knock-down cells. (**A**) Efficiency of *Artd1* and *p65* silencing after RAW 264.7 macrophages transduction with retroviruses carrying shRNA was estimated using real-time PCR. Expression of *Gapdh* was used for normalization. (**B**) Efficiency of *Artd1* and *p65* silencing with shRNA was determined at the level of protein by Western blotting. Tubulin was used as an internal control. (**C**) Experimental design of the in vitro differentiation model (upper panel). Graphic representation of osteoclast formation from RAW 264.7 macrophages; from the left: macrophages without RANKL treatment (-RANKL, day 0 (D0)), 2-day-old (D2) and 3-day-old (D3) osteoclasts (lower panel). (**D**) The resorbing activity of 3-day-old osteoclasts derived from shMOCK and shp65 macrophages were compared based on their capacity to induce matrix dissolution (resorption of bone-mimicking mineral surface) (left panel). The number of matrix dissolution spots was normalized to the number of multinucleated cells (right panel). (**E**) The functional activity of osteoclasts derived from shARTD1 RAW 264.7 macrophages was verified based on their capacity to induce matrix dissolution.

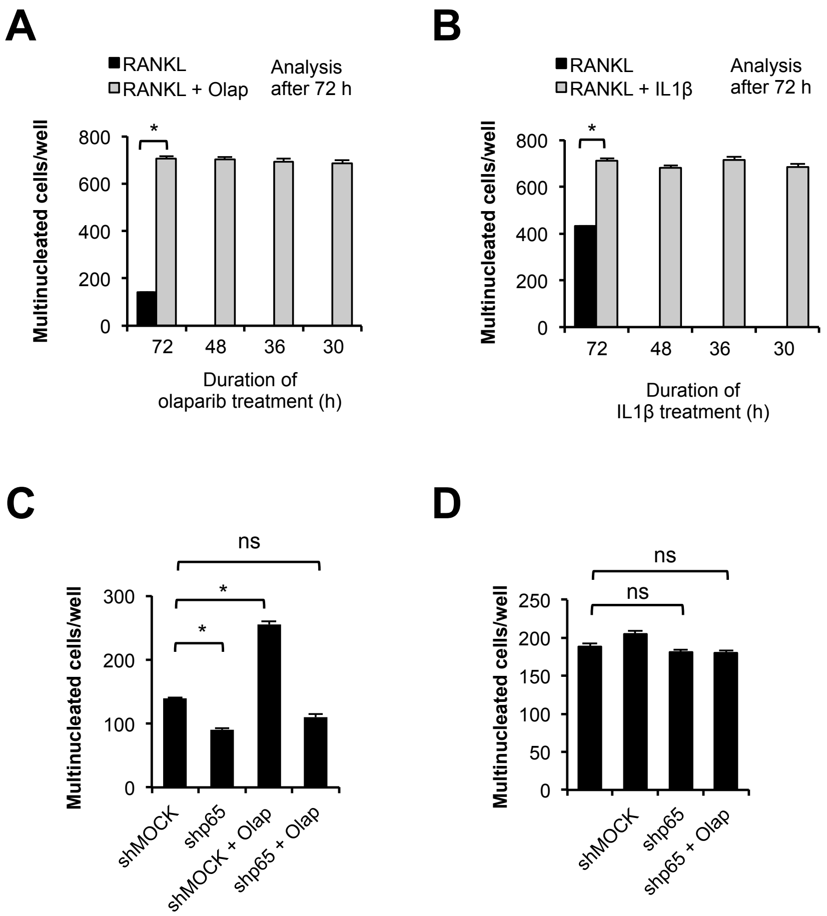

Figure S2. Timing of olaparib or IL-1β effect on multinucleation, and contribution of p65 on olaparib effect on multinucleation. (**A**) Olaparib was administrated at selected time points after induction of osteoclastogenesis, and multinucleated cells were counted after 72 h. (**B**) IL-1β (2 ng/ml) was added at the indicated time points to shMOCK RAW 264.7 macrophages cultured in the presence of RANKL, and multinucleated cells were quantified 72 h after RANKL administration. (**C**) The contribution of p65 in the ADP-ribosylation-mediated increase in osteoclast formation was studied in shMOCK and shp65 RAW 264.7 macrophages induced to differentiate in the presence or absence of olaparib. (**D**) The multinucleation of osteoclasts differentiated in the presence of medium conditioned by shMOCK and shp65 (± olaparib) was compared 48 h after RANKL administration.

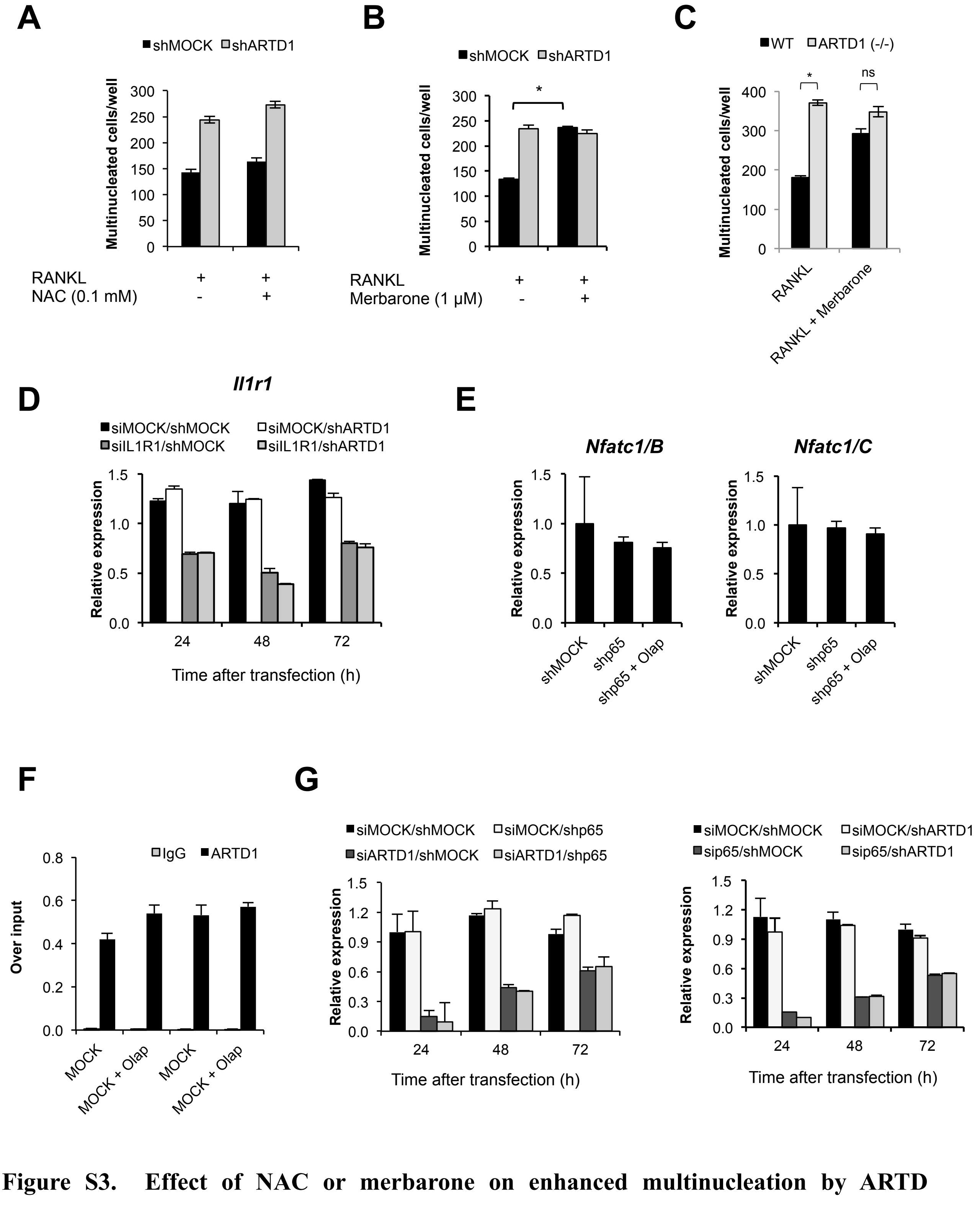

Figure S3. Effect of NAC or merbarone on enhanced multinucleation by ARTD depletion/deletion. (**A**) The ROS scavenger N-acetylcysteine or (**B**) the topoisomerase II inhibitor merbarone were added together with RANKL to shMOCK and shARTD1 RAW 264.7 macrophages and multinucleation was quantified 48 h later. (**C**) The topoisomerase II

inhibitor merbarone was added together with RANKL to the culture of WT and *Artd1* (-/-) osteoclast precursors and multinucleation was quantified 48 h later. (**D**) The recovery of IL-1 receptor 1 (*IL-1r1*) mRNA after silencing with siRNA was determined using real-time PCR. (**E**) The expression of *NAFTc1/B* and *NAFTc1/C* was compared between 3-day-old osteoclasts derived from shMOCK and shp65 (± olaparib) RAW 264.7 macrophages using real-time PCR. (**F**) The association of ARTD1 with chromatin in 3-day-old shMOCK (± olaparib) osteoclasts was determined by ChIP. (**G**) The double *Artd1*/*p65* silencing with siRNA/shRNA over time was monitored with real-time PCR.

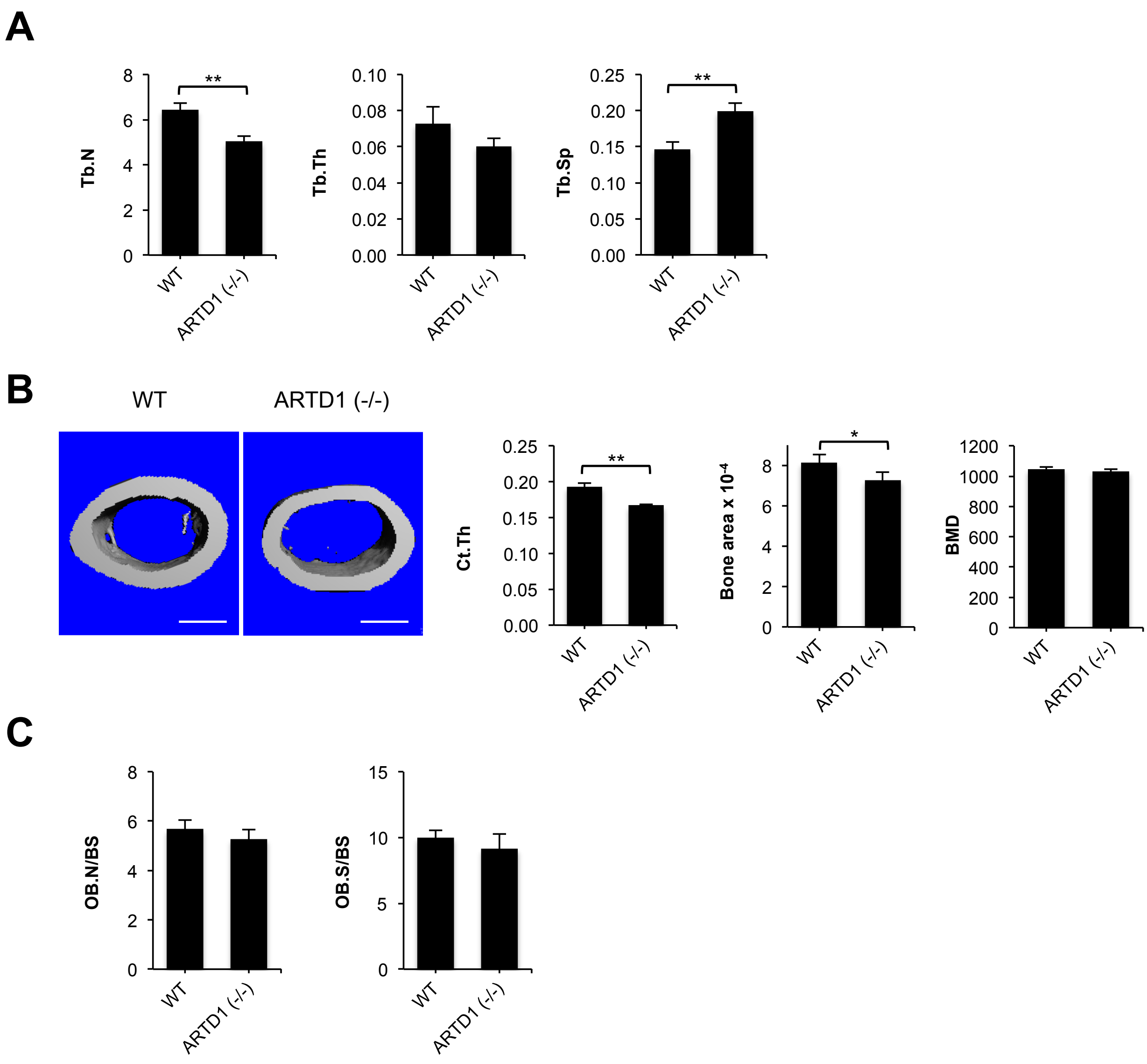

Figure S4. ARTD1 deficiency causes osteopenia without changes in osteoblast parameters. Femurs of 8-week-old *Artd1*-deficient mice (n=4) and their wild-type littermates (n=4) were analysed by μCT. **(A)** Trabecular number (Tb.N), thickness (Tb.Th) or spacing (Tb.Sp). **(B)** Representative 3D reconstruction of mid-shaft cortical bone (left panels, size bars = 500 μm); cortical thickness (Ct.Th), cortical bone area and BMD were measured. **(C)** Osteoblast number/bone surface (OB.N/BS) or surface/bone/surface (OB.S/BS) was quantified from H&E stainings of tibial sections. Values are mean ± SD. * $p<0.05$, ** $p<0.01$.

Supplementary Table

Table S1. List of real-time PCR and ChIP primer sequences

Primers	Sequence
Used for shRNA constructs	
Artd1 Fwd	GATCCCCGGTGATCGGTAGCAACAAATTCAAGAGATTTGTTGCTACCGATCACCTTTTTGGAAA
Artd1 Rev	AGCTTTTCCAAAAAGGTGATCGGTAGCAACAAATCTCTTGAATTTGTTGCTACCGATCACCGGG
p65/RelA Fwd	GATCCCCGAGTTTCAGCAGCTCCTGAACTTCAAGAGAGTTCAGGAGCTGCTGAAACTCTTTTTGGAAA
p65/RelA Rev	AGCTTTTCCAAAAAGAGTTTCAGCAGCTCCTGAACTCTCTTGAAGTTCAGGAGCTGCTAAACTCGGG
Used for *real-time* PCR	
Nfatc1/A Fwd	GGTAACTCTGTCTTTCTAACCTTAAGCTC
Nfatc1/A Rev	GTGATGACCCCAGCATGCACCAGTCACAG
Nfatc1/B Fwd	CCCATCCGCCAGGCTACAGCCGCAGTAA
Nfatc1/B Rev	TTCGGTAAGTTGGGATTTCTGAGTGGTACC
Nfatc1/C Fwd	CCCATCCGCCAGGCTACAGCCGCAGTAA
Nfatc1/C Rev	TGAGTGGTACCAGATGTGGGTCCAGTTTAT
Tracp Fwd	AGATTTGTGGCTGTGGGCGA
Tracp Rev	CTGCACGGTTCTGGCGATCT
Ctsk Fwd	AAGTGGTTCAGAAGATGACGGGAC
Ctsk Rev	TCTTCAGAGTCAATGCCTCCGTTC
Gapdh Fwd	ACTCCACTCACGGCAAATTCA
Gapdh Rev	GGTCTCGCTCCTGGAAGATG
p65/RelA Fwd	GCGAGACCTGGAGCAAGCCATT
p65/RelA Rev	GTGTTGGGGGCCCGGTTATCAA
Artd1 Fwd	GAAGGAAAGAGAAAAGGTGACG
Artd1 Rev	GCAACTCTGTCCAAGATCGCTG
IL-1b Fwd	AAGGAGAACCAAGCAACGACAAAA
IL-1b Rev	TGGGGAACTCTGCAGACTCAAACT
IL-1r1 Fwd	TGGAACAGAGCCAGTGTCAG
IL-1r1 Rev	CAGGAGAAGTCGCAGGAAGT
Rankl Fwd	TGTACTTTCGAGCGCAGATG
Rankl Rev	AGGCTTGTTTCATCCTCCTG

Dspp Fwd	CCTCGGAGGCTTTGAAGACAT
Dspp Rev	CCTCTCGCATGTACCCCATC
Opn Fwd	TCCCTCGATGTCATCCCTGT
Opn Rev	ATCACATCCGACTGATCGGC
Rank Fwd	GCATCCCTTGCAGCTCAACA
Rank Rev	ATGGAAGAGCTGCAGACCAC
Col1a1 Fwd	CGATGGATTCCCGTTCGAGT
Col1a1 Rev	CGATCTCGTTGGATCCCTGG
Used for ChIP	
IL-1b promoter Fwd	CCCCTAAGAATTCCCATCAAGC
IL-1b promoter Rev	GAGCTGTGAAATTTTCCCTTGG
Il6 body Fwd	AAGTGGATTCCCACTGCCACACT
Il6 body Rev	ACAACAGGCAGCATCAGCTGACC
